# Subcutaneous Connective Tissue Reaction to a New Nano Zinc-Oxide Eugenol Sealer in Rat Model 

**DOI:** 10.22037/iej.2017.13

**Published:** 2017

**Authors:** Salma Omidi, Maryam Javidi, Mina Zarei, Siavash Mushakhian, Amirhossein Jafarian

**Affiliations:** a *Department of Endodontics, Dental School, Mazandaran University of Medical Sciences, Sari, Iran; *; b * Dental Material Research Center, Department of Endodontics, Dental School, Mashhad University of Medical Sciences, Mashhad, Iran; *; c * Department of Pathology, Ghaem Hospital, University of Medical Sciences, Mashhad, Iran*

**Keywords:** Biocompatibility, Nanoparticle, Tissue Reaction, Zinc-Oxide Eugenol

## Abstract

**Introduction:**

The aim of this animal study was to evaluate the histological response of the new nano zinc-oxide eugenol (NZOE) sealer in comparison with Pulp Canal Sealer (ZOE based) and AH-26 (epoxy resin sealer).

**Methods and Materials:**

A total of 27 Wistar rats were used. Four polyethylene tubes were implanted in the back of each rat (three tubes containing the test materials and an empty tube as a control). Then, 9 animals were sacrificed at each interval of 15, 30 and 60 days, and the implants were removed with the surrounding tissues.Samples were evaluated for the presence of inflammatory cell (mononuclear cell), vascular changes, fibrous tissue formation and present of giant cell. Comparisons between groups and time-periods were performed using the Kruskal-Wallis and Mann-Whitney U non-parametric tests. The level of significance was set at 0.05.

**Results:**

No significant difference was observed in tissue reactions and biocompatibility pattern of three sealers during 3 experimental periods (*P*<0.05). In all groups the tissue behavior showed tendency to decrease the irritation effect over time. **Conclusion: **The new nano zinc-oxide eugenol sealer has histocompatibility properties comparable to conventional commercial sealers.

## Introduction

Root canal sealers are responsible for the principal functions of the final root filling: sealing off the root canal system, entombment of remaining bacteria and filling the irregularities in the prepared canal [1, 2]. According to Grossman an ideal sealer must be biocompatible and well tolerated by the periradicular tissues [3]. Several, quite different chemical formulations have served as bases for root canal sealers [1]. Unfortunately, the production of sealers that have both good physical and chemical properties and good biological compatibility is difficult. Being well tolerated by tissues, restricts the sealing properties and *vice* *versa *[4, 5]. Zinc-oxide eugenol (ZOE) -based sealers are the oldest used in endodontic therapy. Zinc-oxide is a valuable component of these sealers that is very effective as an antimicrobial agent [6]. Many reforms have been done on this sealers in order to improve their property and also many commercial models are available [6].

Recently nanotechnology has been an ever expanding area of research and opportunity. Due to the novel physical and chemical properties of materials on the nano scale, they have been used to create new products as well as application for life sciences and biotechnology [7]. Nano-technology is also used to produce a large number of dental materials. Advantages of nanoparticles, which have attracted attention in endodontics, are their better penetration into the dental tubules [8], profound antibacterial properties and decreased microleakage [9]. Because of these valuable properties, utilization of nanoparticles in production of endodontic sealers has become favorable for many researchers [5, 10].

Recently, a new endodontic sealer with nano-sized ZO powder particles (NZOE) has been developed in the Dental Material Research Center, Mashhad University of Medical Sciences, Mashhad, Iran. This sealer is similar to various ZOE-based sealers, but with different sizes of ZOE nanoparticles. 

The root obturation materials are in direct contact with dentine and periapical tissues. Hence, the materials should not be cytotoxic and, ideally, be biological stimulator [8]. Therefore, when a new dental material is introduced, its biocompatibility should be determined. Notably despite a considerable amount of research on metallic nanoparticles, their safety is still under discussion. Several biocompatibility tests including cytotoxicity, intraosseous implantations and subcutaneous implantations have been proposed [5, 11]. Although the cytotoxicity of this new formulation to fibroblasts is well documented [5], there is a lack of studies addressing the connective tissue reaction to this endodontic sealer.

The aim of this histopathological animal study was to compare and assess the biocompatibility and connective tissue reaction of this NZOE sealer, a resin-based sealer (AH-26) and a ZOE-based sealer (Root Canal Sealer) by subcutaneous implantation on rats.

## Materials and Methods

The present study was approved by the Animal Research Ethics Committee of the Mashhad University, Iran (Grant No.: 91). A total of 27 male adult Wistar albino rats were used with an average weight of 200 to 220 g. Using blocking technique, the rats were randomly divided in to 3 groups (*n*=9) for 15-day, 30-day and 60-day evaluations.

In this study, in addition to handmade NZOE sealer with particle sizes of 30 nm which was sterilized under UV light for 24 h, as described earlier [12] two commercial sealers, namely AH-26 sealer (Dentsply, De Trey, Konstanz, Germany) and Pulp Canal Sealer (SybronEndo, Orange, CA, USA) were used. 

The animals were anesthetized with an intra-peritoneal injection of a mixture of 47.5 mg/kg of 10% ketamine hydrochloride (Alfasan, Woerden, The Netherlands) and 10 mg/kg of 2% xylazine hydrochloride (Alfasan, Woerden, The Netherlands). Then back of mice were shaved in 4 areas (right front, right rear, left front, left rear) and were disinfected with 10% Betadine (Behsa, Arak, Iran). Then, all the test sealers were prepared according to the user’s manuals and were placed in sterile polyethylene tubes (2.1 mm diameter, 10 mm height) [13]. Then some cuts to a depth of 20 mm were created with #15 surgical blade (Martin, Germany) on the back of the mice in previously prepared and disinfected areas. The skin was denuded with blunt cotton plier. Three tubes carrying different sealers and one empty tube (control) were placed in the prepared cut. Then the edges of the skin was stitched by 0-3 suture (Supa, Tehran, Iran) and the region was disinfected again. To prevent secondary infection, chloramphenicol spray (Vetaque Pharmaceuticals, Sirjan, Iran) was used over the stitches and to help the recovery of animal, 5 cc sugar-salt serum was injected intra-peritoneal. 

All rats were sacrificed in groups after intervals of 15, 30 and 60 days by diethyl ether (Merck, Germany). The areas of the implanted tubes with 1 cm of tissue around the implant were excised and then were fixed in %10 buffered formalin (Merck, Darmstadt, Germany) then they were fixed for 24 h, after which they were processed for paraffin embedding. A series of 4-µm-thick sections were cut parallel to the long axis of the tube and stained with Hematoxylin and Eosin. Tissue reactions, including inflammatory response (mono nuclear cells), formation of fibrous tissue, vascular reactivity, and the presence of giant cells was examined by a trained pathologist who was kept blind based on the grading suggested in the study by Onay *et al.* [14]. The severity of reaction, was classified as follows.

The criteria for scoring the stromal inflammatory response are as follows: *grade 0*; (no reaction), no mononuclear cell infiltration, *grade 1*; (mild reaction), mononuclear cell infiltration comprising<20% of all biopsies, *grade 2*; (moderate reaction), mononuclear cell infiltration comprising 20 to 40% of all biopsies, *grade 3*; (severe reaction), mononuclear cell infiltration comprising >40% of all biopsies.

The criteria for scoring the formation of fibrous tissue are as follow: *grade 0*; (no reaction), normal collagen fiber morphology, *grade 1*; (mild reaction), mild collagen fiber irregularity, *grade 2*; (moderate reaction), moderate collagen fiber irregularity and *grade 3*; (severe reaction), severe collagen fiber irregularity.

The criteria for scoring the vascular changes are as follows: *grade 0*; (no reaction), no significant vascular proliferation, *grade 1*; (mild reaction), the number of vascular structures in one high power field (40×) is <25, *grade 2*; (moderate reaction), the number of vascular structures in one high power field (40×) is between 25 to 50, *grade 3;* (severe reaction), the number of vascular structures in one high power field (40×) is >50.

The presence of giant cells were also scored as present (*grade *1) or absent (*grade *0).

Histopathological evaluation was performed using light Microscope (Olympus CX21, Tokyo, Japan) under 40× and 100× magnification. 

Statistical analysis of tissue inflammatory response for first group was estimated at day 15, second group at day 30 and third group at day 60. Differences among the groups and between the three experimental periods were evaluated using Kruskal-Wallis and Mann-Whitney U non-parametric tests. The level of significance was set at 0.05.

## Results

Macroscopic examination showed satisfactory wound healing in all animals. In all four studied groups, after a period of time, mild to severe inflammation, vascular reactivity, fibrous tissue formation and presence of giant cells were reported. The number and distribution of the implants as well as the severity of tissue reaction are presented in Table 1.

***Day 15: ***There was no significant difference in terms of (inflammation, vascular reactivity, formation of fibrous tissue and the presence of giant cells) between test and control groups (*P*<0.05).

***Day 30: ***Formation of fibrous tissue and giant cells showed no significant differences among four groups. The intensity of inflammatory response (*P*<0.001) and severity of vascular reactivity (*P*<0.01) showed a significant difference between these groups.

Inflammatory response and vascular reactivity were not significantly different among three sealers. Inflammatory response and vascular reactivity were not significantly different between AH-26 and control groups. Intensity of inflammatory response unlike the vascular reactivity between Pulp Canal Sealer and control group (*P*<0.001), as well as NZOE sealer and control group (*P*<0.01) were significant.

***Day 60: ***Formation of fibrous tissue and giant cells showed no significant difference among four groups. There was no significant difference in inflammatory response and vascular reactivity between sealers. Unlike the However, vascular reactivity, inflammatory response was significantly different between Pulp Canal Sealer and control group (*P*<0.01).

Intensity of inflammatory response (*P*<0.01) and vascular reactivity (*P*<0.01) between the control group and AH-26 sealer was significant.

Inflammatory response unlike the vascular reactivity was statistically significant between the NZOE and control group (*P*<0.05).

**Figure1:**
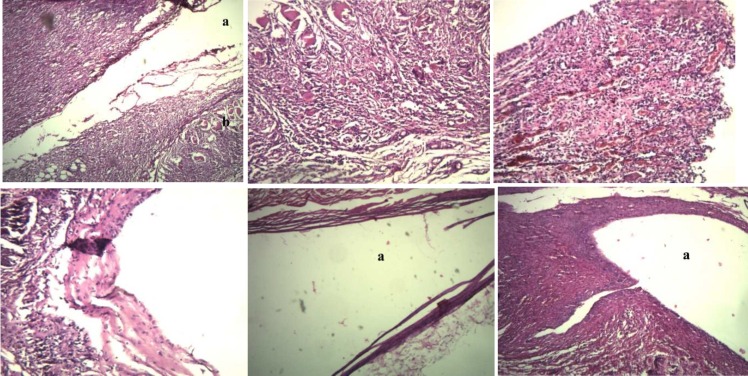
*A*) Day 15; nano ZOE sealer (severe inflammatory reaction and severe giant cell creation) (40×) (a: nano sealer/b: giant cell), *B)* Day 15; Pulpdent (severe inflammation and mild fibrosis) (100×), *C)* Day 15; AH-26 (severe vascular reaction) (100×),* D*) Day 30; nano ZOE sealer (moderate inflammation and fibrosis) (40×), *E)* Day 60, control (without inflammation and mild fibrosis) (a: empty tube) (×40), *F):* nano sealer (mild inflammation and severe fibrosis) (a: nano sealer) (40×)

Overall the severity of tissue inflammatory response induced by all three sealers decreased with time (from day 15 to day 60) and the severity of vascular reactivity increased with time. *P*-value between experimental groups at different time periods are listed in Table 2. The histologic features are shown in Figure 1.

## Discussion

This study was designed for the first time to assess the subcutaneous tissue reaction of a newly developed NZOE sealer in comparison with a commercial ZOE sealer (Pulp Canal Sealer) and an epoxy resin sealer (AH-26). In the present study the inflammatory response (mononuclear cells), vascular reactivity, formation of fibrous tissue and present of giant cells in subcutaneous tissues of rat was evaluated; the results showed that the severity of the tissue reaction decreased with time in all three sealers that is the same as control group. No significant differences were found in the tissue reaction responses among sealers at three time periods. On day 15, inflammatory response (mononuclear cells), vascular reactivity, formation of fibrous tissue and giant cells were similar among the 3 studied sealers and control groups; reactions were moderate to severe that can be caused by surgery trauma [14-16]. On days 30 and 60, tissue reaction reduced around sealers and control group but this reduction in the control group was significantly higher than the rest of the specimens.

Tissue compatibility of filling materials is important due to their contact with periradicular tissues. ZOE-based sealers are amongst the oldest sealers used in endodontics that have been modified for endodontic procedures. Several studies have compared the cytotoxicity and tissue reaction of ZOE-based sealers to other sealers [6, 17-19]. The ZOE sealers with nanoparticles are new. Due to the advances in nano-science in medicine and the benefits of nanostructured materials, the most valuable properties in dentistry is related to its anti-bacterial and better sealer penetration properties [5, 12, 20-22]. 

**Table 1:** Intensity of tissue reaction response at different periods of the study (G=grade

	**Days **	**N**	**Pulpdent**				**AH-26**				**NZOE**			
			**G0**	**G1**	**G2**	**G3**	**G0**	**G1**	**G2**	**G3**	**G0**	**G1**	**G2**	**G3**
**Inflammation (N)**	15	9	0	1	3	5	0	0	2	7	0	1	3	5
	30	9	0	0	7	2	0	6	2	1	1	7	1	0
	60	9	0	3	6	0	0	6	3	0	3	6	0	0
**Vascular change (N)**	15	9	0	7	2	0	0	2	2	5	0	2	3	4
	30	9	0	9	0	0	4	5	0	0	4	5	0	0
	60	9	2	6	1	0	0	9	0	0	6	3	0	0
**Fibrous tissue formation (N)**	15	9	0	5	4	0	2	3	4	0	0	3	6	0
	30	9	0	5	4	0	0	6	2	1	0	4	3	2
	60	9	0	0	7	2	0	1	8	0	0	3	3	3

**Table 2:** *P*-value between experimental groups at different time periods

	**Days **	**Pulpdent**	**Days **	**AH-26**	**Days**	**NZOE**	**Days**	**Control**
**Inflammation (N)**	15 b	0.028	15 a, b	0.001	15 a	0.003	15 a, b	0.001
	30		30 a		30		30 a	
	60 b		60 b		60 b		60 b	
**Vascular change (N)**	15 a, b	0.000	15 a, b	0.000	15 a, b	0.001	15 a, b	0.001
	30 a		30 a		30 a		30 a	
	60 b		60 b		60 b		60 b	
**Fibrous tissue formation (N)**	15 b	0.008	15	0.098	15	0.155	15	0.693
	30 c		30		30		30	
	60 b, c		60		60		60	

a *: Significant difference between 15 and 30 days;*

b *: Significant difference between 15 and 60 days;*

c *: Significant difference between 30 and 60 days*

Since the inflammatory response of connective tissue is similar, subcutaneous implantation studies in animals is one of the most reliable methods to evaluate the biocompatibility of dental materials [17, 23, 24]. In this study, the Wistar rats were used because of their less sensitivity to infection after surgery, being economically viable and available and presenting a plausible model for determining histocompatibility of materials [13]. To ensure standardization and similarity to the clinical situation, polyethylene tubes were used. These tubes are neutral and effectively put the examined materials in contact with the surrounding tissue [17, 24-26]. In this study, the time intervals of 15, 30 and 60 days were used similar to the study by Farhad *et al.* [13]. These ranges were selected to enable to monitor the impact of passage of time on biocompatibility of the sealer. 

Few reports are available in the dental literature about biological testing of nanoparticles [27-31] and until now no study has evaluated the subcutaneous reaction of NZOE sealer. When a new material is introduced, its properties should be investigated and the results must be compared to other conventional materials. Several properties of this new NZOE sealer including antibacterial activity, sealing ability and cytotoxity have been evaluated and its satisfactory results shows that the synthesized pure ZO and ZO mixed with Ag nano powder exhibit better micro-leakage and antibacterial properties in comparison with ZOE and AH-26 sealers [5, 9, 12]. Likewise the biocompatibility of the NZOE sealer on murine fibroblast was comparable to Pulpdent sealer and lower than AH-26. 

Sousa *et al.* [32] evaluated the biological properties of ZOE nanocrystals through intraosseous implantation and reported that the nanocrystals are biocompatible, well tolerated and allow bone formation and remodeling. Barcellos *et al.* [31] concluded that when ZO nanoparticles were added to an adhesive, the cytotoxicity of adhesive was reduced. Memarzadeh *et al.* [30] used ZO nanoparticles as a coating material to inhibit bacterial adhesion and promote osteoblast growth and their findings indicated that NZO can, provide an optimal coating for future bone implants that are both antimicrobial and biocompatible. Several researchers evaluated the biocompatibility of other nanoparticles as new nano-structural calcium silicate systems (CS) and hydroxyapatite (HA-CS) [33], silver nano-particles [7], calcium hydroxide nanoparticles [34] and quaternized polyethylenimine (QPEI) nanoparticles [28, 29]; they reached satisfactory biocompatibility property of nanoparticles. Several studies have evaluated tissue response to endodontic sealers, and most of them have shown that root canal sealers can induce inflammatory reactions when in intimate contact with connective tissues [19, 35-37].

No differences were found regarding the fibrous tissue formation among the groups in each period. This results are supported by Mura *et al.* [17]. Also no differences were found regarding the presence of giant cell reaction among the groups in each period. The multinucleated giant cells, which include the foreign body giant cells (FBGCs) are the dominant early responders to biomaterial implantation and remain at biomaterial-tissue interfaces for the lifetime of the device [38].

The effect of time on obtained results in the present study confirmed the results of previous studies which showed that endodontic sealers can cause tissue damage which decreases with time [2, 13-15, 17, 19]. In *in vivo* studies the moderate and severe inflammation response created by most sealers decreases with time and this event explained the positive role of defending and adaptability of body against foreign substances. 

In all periods tissue reaction caused by nano sealer was somewhat more than the rest of materials which may be related to the physical properties of nanoparticles (owning more contact area with similar volumes) and therefore they can develop more tissue reaction. However this differences were not significant.

A previous study by Molly *et al.* [39] evaluated the biocompatibility of Sealapex, Kerr's sealer, AH-26, and Roth's sealer in a rat model and reported no difference in tissue reaction of the sealers at different time points.

In contrast to the present study, Gomes *et al.* [40] demonstrated that after 30 days, tissue reaction and organization was better in Pulp Canal Sealer (ZOE-based sealer) than Endomethazone and AH-plus. In the survey by Figueired *et al.* [4], the degree of inflammatory response was similar in all experimental groups and decreased over time; Fill canal (ZOE-based sealer) was more toxic than Rickets (ZOE-based-) and AH-26.

In the study by Scarparo *et al.* [36], none of the tested materials (Endorez and Endofil) had ideal properties regarding histocompatibility at intervals of 7, 30 and 60 days and showed more and intense Inflammatory responses. However, in AH-Plus group inflammatory response tended to decrease over time [36].

The conflicting results of the studies can be related to histological effect of endodontic sealers. The difference in the intensity and duration of inflammatory reaction in the several studies might be attributed to the amount of material used, post-implant time, powder/liquid ratio of the sealer and method of survey [17].

Freshly prepared AH-26 is toxic which is attributed to the release of formaldehyde during its chemical setting process [24, 41]. AH-plus is the modified formulation of AH-26 which does not release formaldehyde. However amines which accelerate polymerization in AH-plus composition could be responsible for its initial tissue irritation reported in many studies [24]. In general, fresh resin-based sealers show some toxic effects that decrease over time as the concentration of leachable components is reduced [17].

Eugenol (4-allyl-2-methoxyphenol) is an extract of clove oil which is widely uses in dentistry as a therapeutic agent. Eugenol that leaches out of ZOE-based sealers may participate in the development of periapical inflammation [18]. In the present study, the subcutaneous tissue inflammatory reaction to ZOE-based sealers decreased with time similarly to the result obtained by other researchers [17, 18, 40, 42]. This can be probably due to the neutralization of the eugenol liberated at the start and by the local liberation of corticoids such as dexamethasone and hydrocortisone. Many researchers have suggested that the toxic properties of ZOE-based sealers could be attributed primarily to eugenol and secondarily to zinc ions [18].

## Conclusion

It can be concluded that tissue reactions to the new nano zinc-oxide eugenol sealer had no significant differences with those of AH-26 and Pulp Canal Sealer, conventional sealers. All the implanted materials were well-tolerated by tissues and have acceptable biocompatibility.
